# Construction and Validation of Pyroptosis-Related lncRNA Prediction Model for Colon Adenocarcinoma and Immune Infiltration Analysis

**DOI:** 10.1155/2022/4492608

**Published:** 2022-09-17

**Authors:** Qingsong Liu, Nianzhi Chen, Lu Liu, Qiao Zheng, Wenhao Liao, Maoyuan Zhao, Jinhao Zeng, Jianyuan Tang

**Affiliations:** ^1^Hospital of Chengdu University of Traditional Chinese Medicine, Chengdu, China; ^2^State Key Laboratory of Ultrasound in Medicine and Engineering, College of Biomedical Engineering, Chongqing Medical University, Chongqing, China; ^3^College of Pharmacy, Chengdu University of Traditional Chinese Medicine, Chengdu, China; ^4^Department of Oncology, Hospital of Chengdu University of Traditional Chinese Medicine, Chengdu, China; ^5^TCM Regulating Metabolic Diseases Key Laboratory of Sichuan Province, Hospital of Chengdu University of Traditional Chinese Medicine, Chengdu, China

## Abstract

**Objective:**

Colon adenocarcinoma (COAD) is one of the most prevalent cancers worldwide. However, the pyroptosis-related lncRNAs of COAD have not been deeply examined and validated. Here, we constructed and validated a risk model on pyroptosis-related lncRNAs in COAD.

**Methods:**

The RNA sequencing transcriptome and clinical data of COAD patients were downloaded from The Cancer Genome Atlas (TCGA) database. Differentially expressed pyroptosis-related mRNAs and mRNA-lncRNA coexpression network were identified. After univariate and multifactorial cox analyses of prognosis-related lncRNAs, a risk model was constructed. Next, we analyzed the differences in immune infiltration, immune checkpoint blockade-, immune checkpoint-, and N6-methyladenosine-related gene expressions between the high- and low-risk groups. RT-qPCR was used to validate the expression of lncRNAs.

**Result:**

A risk model was constructed based on 9 pyroptosis-related lncRNAs and separated COAD patients into the high- and low-risk groups. Immune infiltration analysis and immune checkpoint blockade-, immune checkpoint-, and N6-methyladenosine-related genes showed significant differences between the two subgroups. RT-qPCR showed that the 9 pyroptosis-related lncRNAs could be used as prognostic indicators.

**Conclusion:**

A novel risk model based on pyroptosis-related lncRNAs was constructed and demonstrated that these lncRNAs might be used as independent prognostic biomarkers. This will also assist shed light on the COAD prognosis and therapy.

## 1. Introduction

Colon adenocarcinoma (COAD) is one of the most common cancers and the fourth most frequent cause of cancer deaths worldwide [1]. Despite improvements in surgical techniques and adjuvant medical therapy for COAD, the mortality rate is still high [2]. Recently, many molecular prognostic markers and molecular characterization of the tumor have been advocated [3, 4]. Therefore, finding novel prognostic markers and therapeutic targets are essential for preventing and treating COAD.

Pyroptosis is a new kind of discovered programmed cell death, also known as inflammatory necrosis, which is characterized by cell rupture and death that releases inflammatory mediators and activates a solid inflammatory response [5, 6]. The inflammatory reaction caused by pyroptosis improves the tumor immune microenvironment. It promotes the immune response of CD8+ T cells, which stimulate strong antitumor immunity and achieve a synergistic antitumor effect with immune checkpoint inhibitors [7]. Notably, a recent study examined whether pyroptosis promoted the development of COAD and revealed a previously undiscovered link between pyroptosis and COAD tumorigenesis, which provided a new research field into the pathogenesis of COAD combined with pyroptosis [8]. Therefore, it is vital to investigate how pyroptosis participates in the pathogenesis of COAD. Elucidating the molecular mechanisms underlying COAD combined with pyroptosis is essential to reveal the predictive potential of pyroptosis-related genes and their association with the immune state.

Long noncoding RNAs (lncRNAs) activate several inflammasomes, resulting in cell pyroptosis [9]. lncRNAs play essential roles in a wide range of biological processes and are involved in the complex mechanism of colorectal carcinogenesis [10]. lncRNA GAS5 and lncRNA HOXD-AS1 inhibit the progression of COAD and metastasis [11, 12]. However, limited studies have focused on pyroptosis-related lncRNAs in COAD.

**Figure 1:**
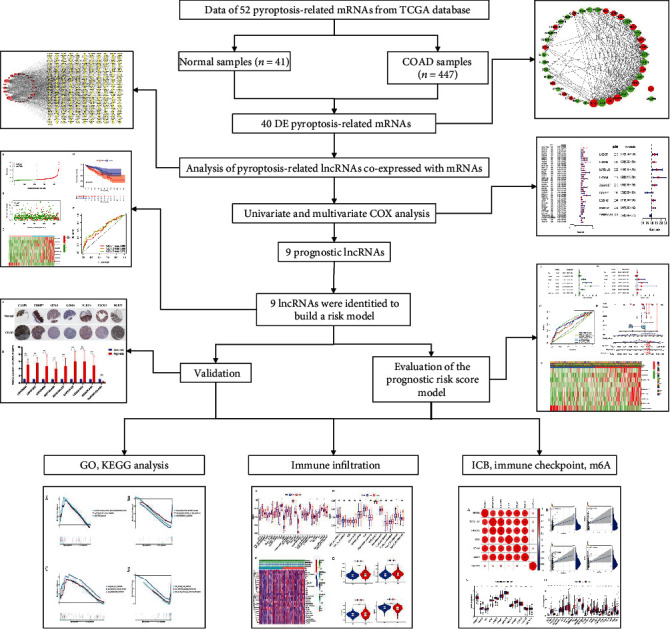
The flowchart of this study.

In this study, we used public datasets to develop and verify a COAD prognostic signature based on pyroptosis-related lncRNAs. In addition, we analyzed the differences in immune infiltration, immune checkpoint blockade-, immune checkpoint-, and N6-methyladenosine- (m6A-) related gene expression between the high- and low-risk groups. In short, we have established a risk model to predict COAD patients' prognosis and has potential clinical application value. The flow chart of our research is shown in Figure 1.

## 2. Materials and Methods

### 2.1. Data Collection and Processing

The RNA transcriptome data and clinical data of 447 COAD patients were obtained from The Cancer Genome Atlas (TCGA) database (https://portal.gdc.cancer.gov/) on September 12, 2021. Htseq-counts were used as the sequencing data formats. Patients with incomplete survival information and clinical data were excluded from further evaluation. Then, the data were compiled and annotated to protein-coding genes and lncRNAs using the Ensembl human genome browser (http://asia.ensembl.org/info/data/index.html) using the Perl program.

### 2.2. Identification of Pyroptosis-Related Differentially Expressed Genes

The previous studies [13, 14] showed that 52 genes were defined as pyroptosis-related regulators. The 52 pyroptosis-related mRNAs between 41 normal and 447 COAD were extracted from TCGA. We were using the “limma” package to identify pyroptosis-related differentially expressed mRNAs (PDMs) with *p* < 0.05. A protein-protein interaction (PPI) network with a threshold ≥ 0.4 was downloaded using the STRING database (http://string-db.org/). Cytoscape software (version 3.8.2) was used to visualize the PPI network.

### 2.3. Acquisition of Pyroptosis-Related lncRNAs Related to COAD Prognosis

We constructed an mRNA-lncRNA coexpression network with |Pearson correlation coefficient| > 0.4, and the threshold was set to *p* < 0.001. Visualization analysis was using Cytoscape software to exhibit the coexpression relationship between PDMs and lncRNAs. After the univariate and multivariate Cox regression analyses with the threshold of *p* < 0.05, the pyroptosis-related lncRNAs associated with prognosis were acquired.

### 2.4. Construction of the Prognostic Risk Model Based on Pyroptosis-Related lncRNAs

We used the pyroptosis-related lncRNAs to construct the prognostic risk model. The risk score was calculated for each COAD patient using the following formula: risk score = *Σi* coefficient (lncRNA1) × expression (lncRNA1) + coefficient (lncRNA2) × expression (lncRNA2) + ⋯⋯+coefficient (lncRNA*n*) × expression (lncRNA*n*). The patients were divided into the high-risk and low-risk groups according to the median value of the risk score.

### 2.5. Evaluation of the COAD Prognostic Model

Survival probability was determined using Kaplan-Meier between the high-risk and low-risk groups, and the ROC was used to predict the sensitivity and specificity of the risk model and calculate the AUC of the overall survival (OS) rate of 1-year, 3-year, and 3-year COAD. Next, Cox regression was utilized to establish whether the risk ratings were a reliable predictor of COAD. Additionally, a nomogram with the 1-year, 2-year, and 3-year survival rates was constructed using the “rms” package of R software.

### 2.6. Gene Set Enrichment Analysis (GSEA)

To further clarify the biological mechanism and signaling pathway differences between the two risk groups, Gene Ontology (GO) and Kyoto Encyclopedia of Genes and Genomes (KEGG) pathway enrichment analyses were performed using Gene Set Enrichment Analysis (GSEA, http://www.gsea-msigdb.org/gsea/msigdb/annotate.jsp).

### 2.7. Immune Infiltration Analysis

The infiltration of 16 immune cells and the activities of 13 immune-related pathways were evaluated using single-sample gene set enrichment analysis (ssGSEA) using the “gsva” R package. We used the “estimate” package to evaluate the components in the TME of COAD between the high-risk and low-risk groups, and the components included three scores: the ESTIMATE score, immune score, and stromal score. We also used the CIBERSORT R package (https://cibersort.stanford.edu/) to calculate the fraction of immune infiltrating cells in COAD samples between the two risk groups. The results of CIBERSORT were screened at *p* < 0.05. We evaluated which type of tumor-infiltrating immune cells in the TME of COAD was associated with the risk score by comparing the differences in each type of immune infiltrating cell between the high-risk and low-risk groups.

### 2.8. Immune Checkpoint Blockade-, Immune Checkpoint-, and m6A-Related Gene Analysis

To assess the relationship between the risk scores based on pyroptosis-related lncRNAs associated with immune checkpoint blockade (ICB) genes, we correlated the expression of six critical genes for immune checkpoint blockade therapy with the risk score of the pyroptosis-related lncRNA signature. The six essential genes included CD274, PDCD1LG2, PDCD1, CTLA-4, IDO1, and HAVCR2. We also constructed boxplots using the “ggplot2” R package to visualise the correlation of the risk score with the expression of immune checkpoint- and m6A-related genes between the high-risk and low-risk groups considering the potential for immunotherapy.

### 2.9. RT-qPCR Analysis of Human Colon Adenocarcinoma Tissues

Sixteen COAD samples were collected from patients after surgical excision at the Hospital of Chengdu University of Traditional Chinese Medicine. The specimens were snap-frozen in liquid nitrogen and stored at -80°C until they were analyzed. We divided the patients into the high-risk and low-risk groups to validate the reliability of the prognostic model of pyroptosis-related lncRNAs. This study was approved by the Ethics Committee of the Hospital of Chengdu University of Traditional Chinese Medicine (approval no. 2020KL-062), and informed consent was obtained from all participants.

Following the manufacturer's instructions, we extracted RNA from colon tissues using TRIzol reagent (Life Technologies CA, USA). We randomly assigned the RNA samples from each COAD tissue extracted for RT-qPCR analysis. Reverse transcription was performed using the SureScript First-Strand cDNA Synthesis kit (GeneCopoeia, Guangzhou, China) at 45°C for 1 hour. The RT-qPCR analysis was performed using BlazeTaq SYBR Green qPCR master mix (GeneCopoeia, Guangzhou, China) and Applied Biosystems 7500 Fast Real-Time PCR System (Applied Biosystems). The primer sequences for the nine pyroptosis-related lncRNAs are shown in Supplementary table 1. We used the 2^ΔΔCt^ values to calculate the expression level of each gene in every sample, and all RNAs of every sample were analyzed in three independent experiments.

In addition, protein expression levels of 10 pyroptosis-related genes in COAD tissues and normal tissues were compared according to the staining intensity and percentage of stained cells in the tissues from The Human Protein Atlas (https://www.proteinatlas.org/) database.

### 2.10. Statistical Analysis

R software version 4.0.4 was used to analyze the data. Overall survival based on the risk model was evaluated using Kaplan-Meier survival analysis. We performed univariate and multivariate Cox regression analyses to identify the prognostic value of the risk score compared with clinical characteristics. A nomogram was constructed by combining the clinical characteristics of COAD patients and the risk score. The Wilcoxon test was used to analyze the differences in immune cells and the expression of the immune checkpoint-, m6A-, and IBD-related genes. The RT-qPCR data were analyzed by *t*-test with GraphPad Prism software (version 9.0). ^∗^*p* < 0.05, ^∗∗^*p* < 0.01, and ^∗∗∗^*p* < 0.001 were considered significant.

## 3. Results

### 3.1. Identification of Pyroptosis-Related mRNAs in Normal and COAD Tissues

**Figure 2:**
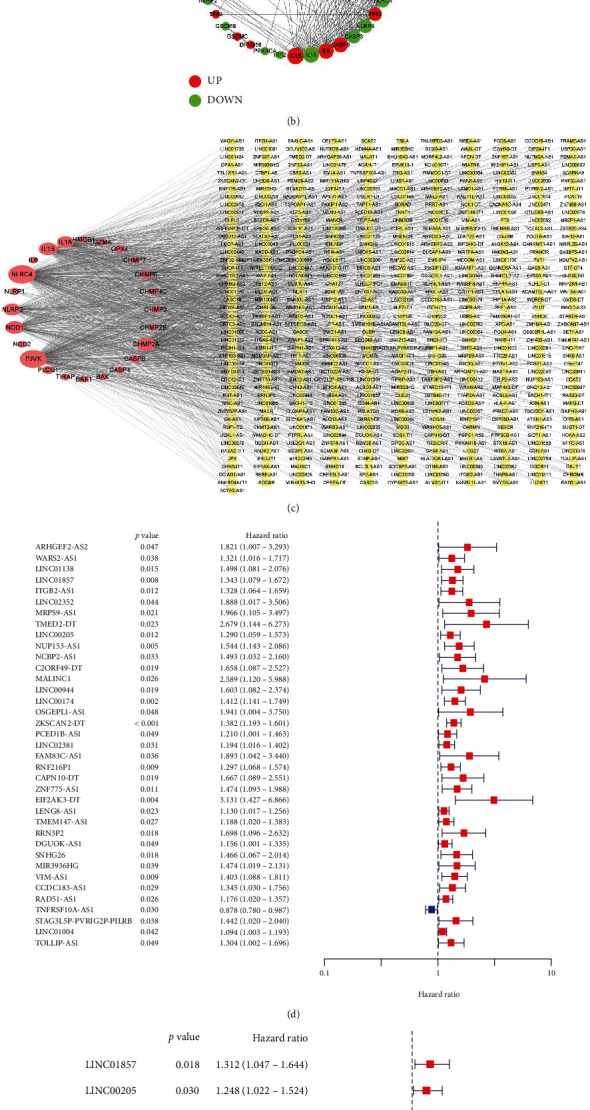
Identification of pyroptosis-related lncRNAs with prognostic significance. (a) A heat map of the expression level of 40 differential pyroptosis-related mRNAs between the normal (blue) and COAD tissues (red). ^∗^*p* < 0.05, ^∗∗^*p* < 0.01, and ^∗∗∗^*p* < 0.001. (b) Construction of a PPI network to visualise the interactions of the pyroptosis-related mRNAs. (c) Coexpression network of pyroptosis-related mRNAs-lncRNAs. Yellow represented lncRNAs. Pink represented mRNAs. (d) 37 pyroptosis-related lncRNAs were screened by univariate Cox analysis. (e) Nine pyroptosis-related lncRNAs were screened by multivariate Cox analysis. (f) Sankey diagram showing the associations between pyroptosis-related lncRNAs, mRNAs, and risk type.

The expression levels of 52 mRNAs in the TCGA dataset from 41 normal and 447 COAD tissues were compared. 40 mRNAs exhibited significantly different expression patterns (*p* < 0.05) (Figure 2(a)). 22 mRNAs were downregulated, and 18 mRNAs were upregulated. A PPI network visualizes the interactions between genes (Figure 2(b)).

### 3.2. Coexpression Network of Pyroptosis-Related mRNAs and lncRNAs with Prognostic Value

A coexpression network containing 26 PDMs and 1186 lncRNAs was constructed using Cytoscape (Figure 2(c)). Subsequently, 1186 lncRNAs were analyzed using univariate Cox analysis, and 37 lncRNAs were screened (Figure 2(d)). These 37 lncRNAs were further analyzed using multivariate Cox analysis (Figure 2(e)) and obtained 9 lncRNAs with prognostic significance. A Sankey diagram was constructed to visualize two lncRNAs with protective factors (CAPN10-DT and TNFRSF10A-AS1) and seven lncRNAs with risk factors (LINC01857, LINC00205, NUP153-AS1, LINC00944, ZKSCAN2-DT, DGUOK-AS1, and LENG8-AS1) (Figure 2(f)).

### 3.3. Construction of a Risk Model Based on Pyroptosis-Related lncRNAs

Multivariate Cox analysis calculated the risk score with the coefficients of nine lncRNAs in the risk model. The following risk score formula was used: CAPN10 − DT × (−0.617242409511103) + DGUOK‐AS1 × (0.179614289060558) + LINC01857 × (0.2717126160098) + LINC00205 × (0.221328820499054) + NUP153 − AS1 × (0.407383818674744) + LINC00944 × (0.533137375733077) + ZKSCAN2‐DT × (0.266203945737456) + LENG8‐AS1 × (0.164284611822428) + TNFRSF10A‐AS1 × (−0.157784377489306). The median risk score was used as the threshold value according to each calculated risk score of every patient. The 447 COAD patients were divided into a low-risk group (*n* = 224) and high-risk group (*n* = 223).

**Figure 3:**
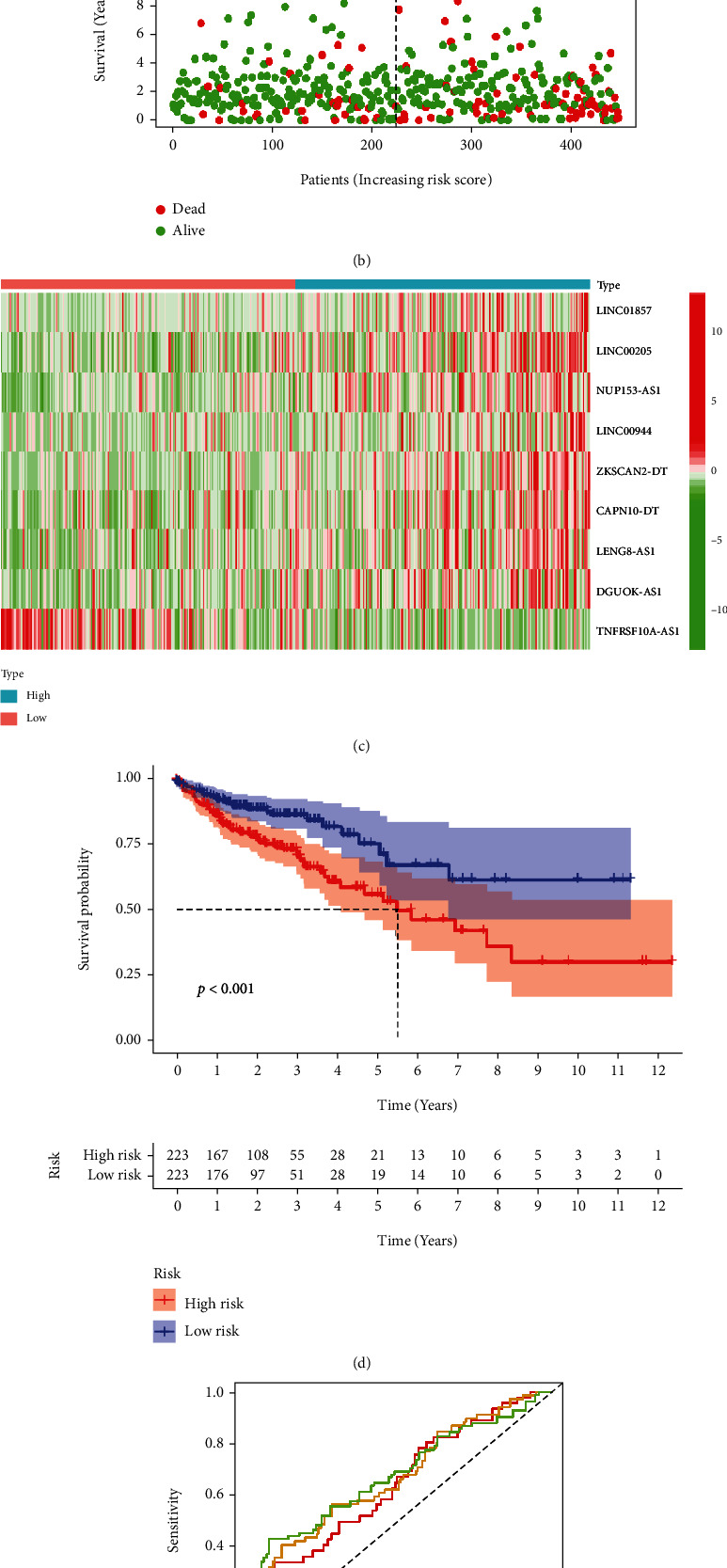
Prognostic value of the risk model based on the 9 pyroptosis-related lncRNAs. (a) The risk curve was based on the risk score for each COAD patient. Red indicates high risk, and blue indicates low risk. (b) A scatterplot based on the survival status of each sample of the risk model. Green and red dots indicate alive and death. (c) The expression of 9 pyroptosis-related lncRNAs in the high-risk and low-risk groups. (d) The Kaplan-Meier curves predict COAD patients' survival probability between the high-risk and low-risk groups (*p* < 0.001). (e) The ROC curves of the risk model at 1, 2, and 3 years.

To evaluate the overall prognostic value of this risk model based on pyroptosis-related lncRNAs, we determined the survival status and risk score distribution, shown in Figures 3(a)–3(c). The survival rate of the high-risk group was significantly worse than the low-risk group, and COAD patients with higher risk scores tended to die earlier (Figure 3(c)).

The Kaplan-Meier survival curves showed that the survival probability of COAD patients in the high-risk group was significantly lower than that in the low-risk group (*p* < 0.001) (Figure 3(d)).

The ROC curve identified the risk score with significant predictive sensitivity and specificity. The area under the curve (AUC) was calculated, and the AUCs at 1, 3, and 5 years were 0.640, 0.666, and 0.676, respectively (Figure 3(e)).

### 3.4. Independent Prognostic Value of the Risk Model

**Figure 4:**
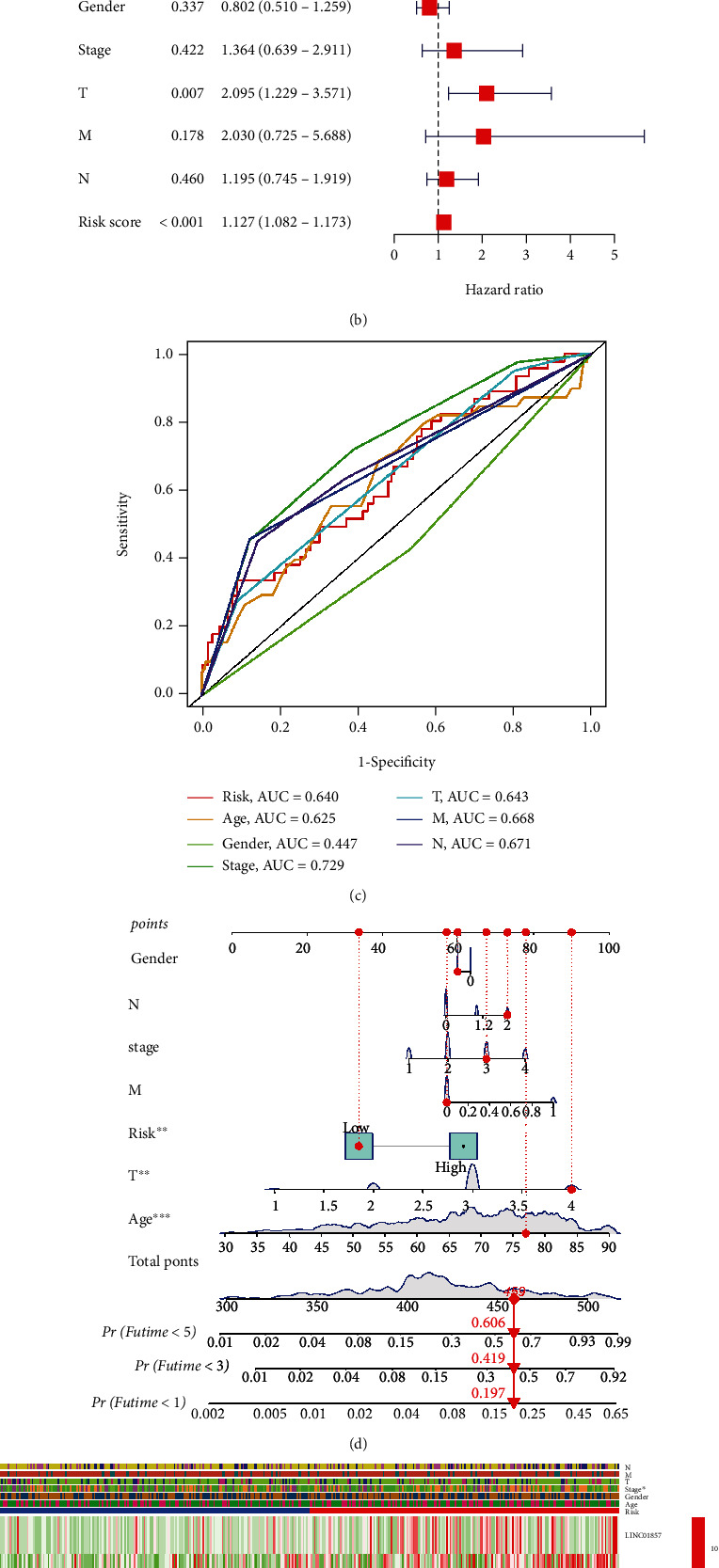
Correlation of the 9 pyroptosis-related lncRNA signatures with clinical features. (a) Univariate and (b) multivariate Cox regression analyses of clinical characteristics and risk score from this risk model by a measure of the hazard ratio. (c) The ROC curve analyses for determining the sensitivity and specificity of this signature by comparing risk score, age, gender, stage, and TNM stage. (d) The clinical prognostic nomogram was developed to predict the 1-year, 3-year, and 5-year survival of COAD patients between the high-risk and low-risk groups. (e) A heat map visualized the expression of the 9 pyroptosis-related lncRNAs in two risk groups and combined with COAD clinical features (age, gender, TNM stage, and stage).

The risk score in this risk model was identified as an independent prognostic factor using univariate Cox regression (HR = 1.120, 95% CI: 1.079-1.163) and multivariate Cox regression (HR = 1.127, 95% CI: 1.082-1.173) combined with clinicopathological characteristics in COAD patients (Figures 4(a) and 4(b)).

The ROC analyses also predicted the sensitivity and specificity of the risk model compared with a risk score, age, sex, clinical stage, and TNM stage (Figure 4(c)), and the AUC was 0.64.

A clinical prognostic nomogram was developed to predict 1-year, 3-year, and 5-year survival (Figure 4(d)). We found that the low-risk group's 1-year, 3-year, and 5-year survival rates with total points were 80.3%, 58.1%, and 39.4%.

We generated a heat map to visualize the distribution of the 9 pyroptosis-related lncRNAs in the two risk groups combined with clinical features, including age, sex, and TNM stage (Figure 4(e)).

### 3.5. Gene Set Enrichment Analysis

**Figure 5:**
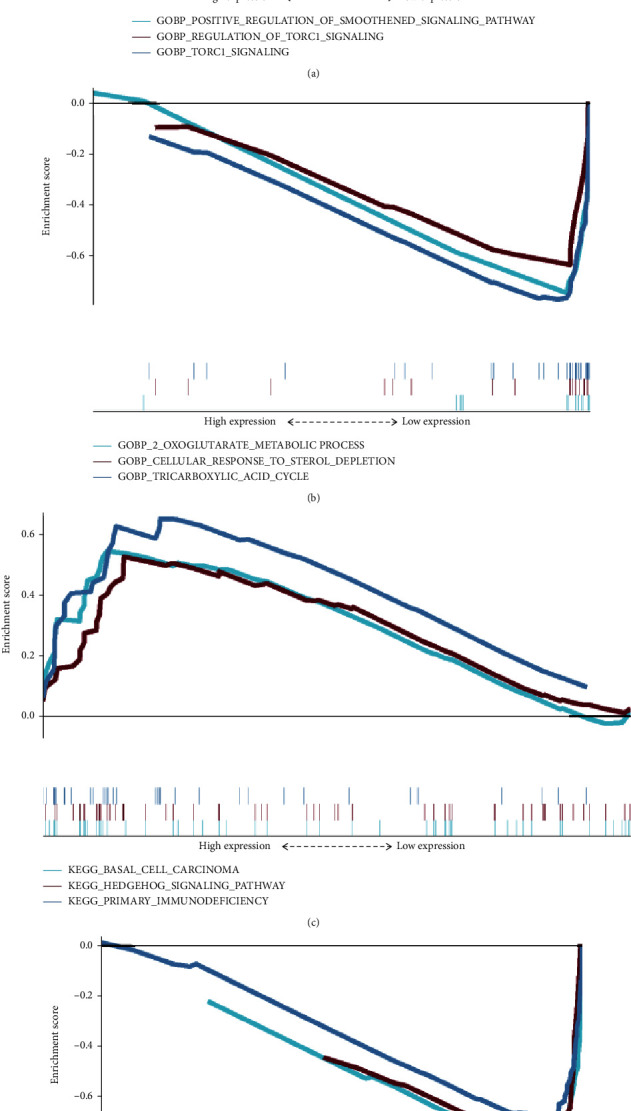
GO and KEGG analyses. (a, b) GSEA results show differential gene enrichment in GO-BP with (a) high-risk and (b) low-risk groups of pyroptosis-related lncRNAs. (c, d) GSEA results show differential gene enrichment in KEGG with the (c) high-risk group and (d) low-risk group of pyroptosis-related lncRNAs.

The GO-BP results showed that these prognostic genes were strongly associated with TORC1 signalling (NES = 2.1856544), regulation of TORC1 signalling (NES = 2.1721838), and regulation of smoothened signaling pathway (NES = 2.1621225) were enormously enriched in the high-risk group (Figure 5(a)). In the low-risk group, the tricarboxylic acid cycle (NES = −2.1532452), 2-oxoglutarate metabolic process (NES = −2.030756), and cellular response to sterol depletion (NES = −1.988547) were enriched (Figure 5(b)).

KEGG analysis in the high-risk group identified basal cell carcinoma (NES = 1.8805724), hedgehog signaling pathway Hedgehog (NES = 1.85883), and primary immunodeficiency (NES = 1.8171213) (Figure 5(c)). With the low-risk group, citrate cycle TCA cycle (NES = −2.196363), valine-leucine-isoleucine degradation (NES = −2.05986), and terpenoid backbone biosynthesis (NES = −2.0022373) were performed (Figure 5(d)).

### 3.6. Correlation between the Risk Score and the Tumor Immune Environment

**Figure 6:**
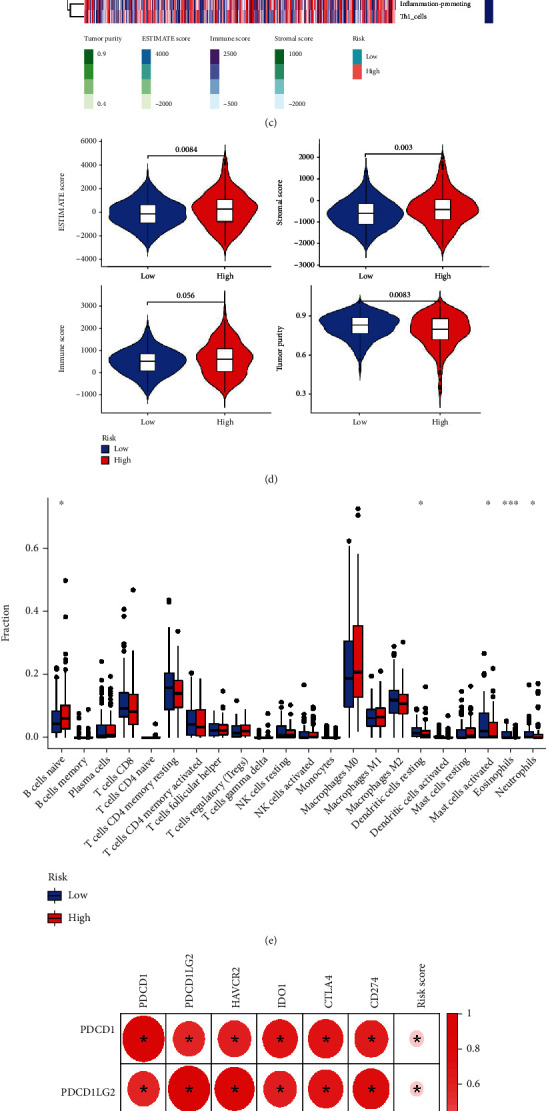
Altered expression between the low-risk and high-risk groups in terms of immune infiltration, immune checkpoint blockade, immune checkpoint analysis, and N6-methyladenosine-related gene analysis. (a) The ssGSEA scores were compared by 16 types of immune cells. (b) 13 immune-related pathways between the low-risk (blue) and high-risk (red) groups. (c) A heat map to evaluate the components in the TME of COAD between the high-risk and low-risk groups by ESTIMATE. A higher score indicated a larger ratio of the component in the TME. (d) ESTIMATE score, Stromal score, immune score, and tumor purity between two risk groups. (e) The fraction of immune infiltrating cells between two subgroups by CIBERSORT. (f) Correlation analyses the expression of 6 key genes of immune checkpoint blockade with the pyroptosis-related lncRNA signature risk score. The differential expression of (g) immune checkpoints and (h) N6-methyladenosine-related genes between the high-risk and low-risk groups. ^∗^*p* < 0.05, ^∗∗^*p* < 0.01, and ^∗∗∗^*p* < 0.001.

The results from ssGSEA showed that the risk score was significantly associated with four types of immune cell infiltration, such as HLA, T helper cells, Th2 cells, and the type I IFN response (Figure 6(a)). The responses of the HLA and the type I IFN were related to the risk model (Figure 6(b)).

The ESTIMATE was used to assess the low-risk and high-risk groups' stromal, immune, and ESTIMATE scores (Figure 6(c)). Specific differences were observed in the immune score (*p* = 0.056). They showed significant differences in the ESTIMATE score (*p* = 0.0084), stromal score (*p* = 0.003), and tumor purity (*p* = 0.0083) between the two groups with higher risk scores (Figure 6(d)).

The CIBERSORT analysis found that 5 cells correlated with prognostic characteristics: naïve B cells, resting dendritic cells, activated mast cells, eosinophils, and neutrophils (Figure 6(e)). The results showed that more immune cells infiltrated the high-risk group. This may be further elucidated to develop tumor immunotherapy in COAD.

### 3.7. ICB-, Immune Checkpoint-, and m6A-Related Gene Analysis

Unprecedented advances have been made in cancer treatment with the use of ICB. We analyzed the correlation between ICB and prognostic characteristics based on the pyroptosis-correlated lncRNA signature and revealed the potential risk characteristics in ICB treatment of COAD (Figure 6(f)).

Due to the potential of checkpoint inhibition in immunotherapy, we further examined differences in the expression of immune checkpoint-related genes between the two risk groups. The results showed that approximately 25 immune checkpoint-related genes significantly differed between the high-risk and low-risk groups. We observed the expression of all immune checkpoint-related genes between the two subgroups, most of which were higher in the high-risk group, and only TNFSF9 was higher in the low-risk group (Figure 6(g)).

m6A is involved in regulating some tumor-targeted therapy genes [15]. We investigated the expression of m6A-related genes between the two risk groups, and the results showed that a total of 6 m6A-related genes were significantly different between the high-risk and low-risk groups, and RBM15, FTO, YTHDF1, METTL3, and YTHDC1 expressions in the high-risk group were significantly higher than the low-risk group. In contrast, the expression of YTHDF2 in the low-risk group was higher (Figure 6(h)).

### 3.8. Validation of the Risk Model

**Figure 7:**
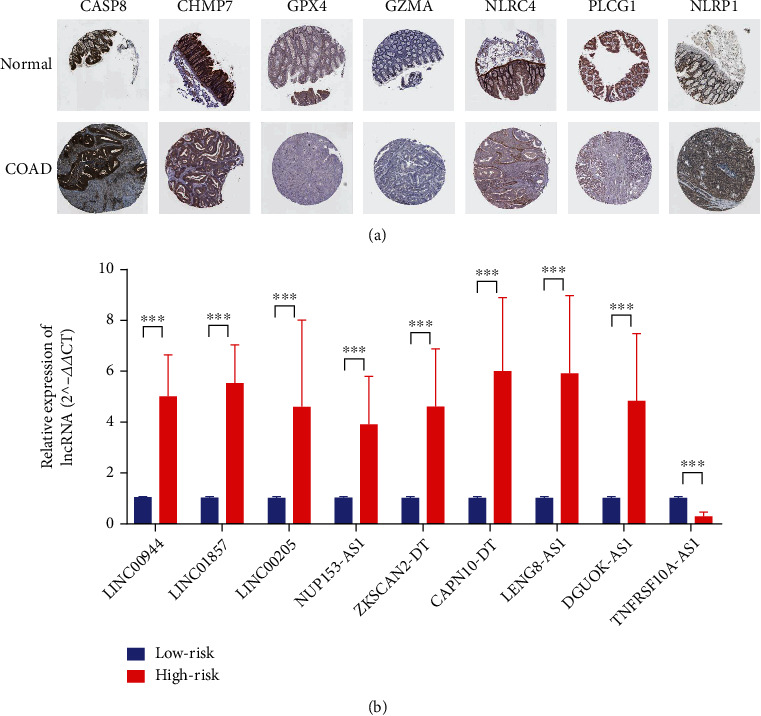
The validation of pyroptosis-related genes in COAD tissues. (a) Immunohistochemical images of pyroptosis-related gene protein expression levels in the HPA database. (b) The expression of 9 pyroptosis-related lncRNAs between the high-risk and low-risk groups by RT-qPCR. ^∗^*p* < 0.05, ^∗∗^*p* < 0.01, and ^∗∗∗^*p* < 0.001.

The immunohistochemical staining images were analyzed in the HPA database to observe the expression levels of pyroptosis-related mRNA proteins in COAD. HPA database does not include NOD1, CHMP7, and PJVK. The protein expression levels of 7 pyroptosis-related genes in COAD tissues and normal tissues were obtained (Figure 7(a)).

We performed RT-qPCR to validate the expression of the nine pyroptosis-related lncRNAs in our risk model. The results showed that all 9 lncRNAs were differentially expressed between high-risk and low-risk COAD tissues (Figure 7(b)). The TNFRSF10A-AS1 was decreased, while the other eight lncRNAs were increased in the high-risk group. Supplementary Figure 1 shows the expression of 9 pyroptosis-related lncRNAs between the high-risk and low-risk groups in human COAD tissues by RT-qPCR (delta Ct).

## 4. Discussion

Pyroptosis is a proinflammatory programmed cell death distinct from noninflammatory apoptosis and depends on the cleavage of the gasdermin family protein GSDMD by the inflammatory caspase protease [16–18]. In the age of immunotherapy, all tumors may be divided into three types according to the antitumor immune response: immune-inflamed tumors, immune-excluded tumors, and immune-deserted tumors [19]. Immune-inflamed tumors are known as “hot tumors,” and many immune infiltrating cells exist in this type of tumor. Therefore, the effect of immunotherapy using immune checkpoint inhibitors is better. However, in immune-excluded and immune-deserted tumors, also known as “cold tumors,” the effect of immunotherapy is not ideal due to poor immune cell infiltration. Unprecedented advances from important research confirmed that pyroptosis improved the effect of immunotherapy. Many tumor cells trigger pyroptosis, which stimulates an inflammatory reaction, improves the tumor immune microenvironment, activates the antitumor immune response, and achieves a synergistic antitumor effect with immune checkpoint inhibitors [20, 21].

Several recent studies have proposed prognostic models for COAD [22–24]. However, the studies' predictive performances have not been validated in COAD, and the differences in immune infiltrating cells and immune checkpoint blockade-, immune checkpoint-, and m6A-related genes between the high and low risks of the prediction model were not analyzed. In this study, we constructed and verified a COAD prognostic signature based on pyroptosis-related lncRNAs.

The present study investigated pyroptosis-related lncRNAs in COAD patients and constructed a risk model that included 9 lncRNAs (LINC01857, LINC00205, NUP153-AS1, LINC00944, ZKSCAN2-DT, DGUOK-AS1, LENG8-AS1, CAPN10-DT, and TNFRSF10A-AS1).

LINC01857 is involved in gastric cancer, glioma, diffuse large B-cell lymphoma (DLBCL), hepatocellular carcinoma (HCC), and breast cancer (BC). LINC01857 acts as an oncogene that promotes BC development by promoting H3K27Ac and CREB1 transcription [25], regulates glioma progression by modulating the miR-1281/TRIM65 pathway [26], promotes the proliferation of cancer cells by activating the PI3K/mTOR pathway, and facilitates the EMT process in DLBCL [26]. It is associated with metastasis and poor prognosis in gastric cancer [27], and overexpression of LINC01857 in HCC promotes cell proliferation by regulating AGR2 and upregulating the AKT and ERK pathways [28]. Several bioinformatics studies also confirmed the prognostic role of LINC01857 in cancer [29, 30].

The same characteristics also occurred in LINC00205. It may be used as a novel prognostic indicator of several cancers in multiple bioinformatics analyses, such as glioma [31], gastric cancer [32], and hepatocellular carcinoma [33–35]. Multiple lines of experimental evidence from basic research demonstrated the oncogenic role of LINC00205 in HCC, which may be beneficial for diagnosing and treating HCC [34, 36]. LINC00205 also facilitates malignant phenotypes and may be a target for lung cancer [37].

Clinicopathological and experimental evidence indicates that LINC00944 plays a role as an oncogene in renal cell carcinoma (RCC) [38] and has prognostic value in breast cancer [39]. Other research and RT-qPCR validation showed that TNFRSF10A-AS1, a protective risk factor in our risk model, was associated with autophagy and contributed to poor colon adenocarcinoma prognoses [40]. Research on the lncRNA DGUOK-AS1 is currently focused on cervical and breast cancer. Studies revealed the significant roles of DGUOK-AS1 as a prognostic predictor [41], and experiments identified the detailed mechanism of progression, migration, and angiogenesis in BC [42], and the mechanism by which its overexpression promoted cervical cancer progression [43, 44].

Few studies investigated the four lncRNAs, NUP153-AS1, ZKSCAN2-DT, LENG8-AS1, and CAPN10-DT. This evidence indicated the prognostic value, diagnostic value, and potential role of the 5 pyroptosis-related lncRNAs (LINC01857, LINC00205, LINC00944, DGUOK-AS1, and TNFRSF10A-AS1) as therapeutic targets for discovering novel strategies in multiple tumors. They may also have common values in COAD. However, there is no current research in COAD or specific molecular mechanisms based on these pyroptosis-related lncRNAs in COAD. Our research provides novel perspectives for further exploration in this field. More experiments are needed to validate the specific mechanism and role of these lncRNAs in the progression and tumorigenesis of COAD and their correlation with pyroptosis. Notably, although the remaining four identified pyroptosis-related lncRNAs (NUP153-AS1, ZKSCAN2-DT, LENG8-AS1, and CAPN10-DT) have not been well studied, these lncRNAs maintained the most intimate links with many pyroptosis-related genes that were deeply studied and confirmed the role of pyroptosis.

Our study demonstrated that the pyroptosis-related lncRNA signature was to infiltrations of eosinophils, neutrophils, and resting dendritic cells, indicating the crosstalk between these pyroptosis-related lncRNAs and immune cells. Eosinophil was a critical driver of antitumor immunity via the activation of type 1 T cell and CD8+ T responses [45], and it was a prognostic indicator of COAD [46]. Neutrophil infiltration combined with TGF*β* activation in the TME suppresses the immune mechanism and facilitates tumorigenesis of COAD [47]. Dendritic cells initiate the subsequent stage of immunity and play a key role in tumor immunotherapy [48]. These three types of immune cells are related to pyroptosis-related lncRNAs in our research, and it is possible to explore the potential of these lncRNAs in activating the immune response and how to improve the tumor microenvironment in the future.

We associated four key immune checkpoint inhibitor genes (PDCD1, CD274, PDCD1LG2, CTLA-4, HAVCR2, and IDO1) and 25 immune checkpoint-related genes with risk scores with revealing the potential therapeutic targets in the treatment of COAD. These candidate pyroptosis-related lncRNAs and genes may activate pyroptosis in tumor cells, target immune checkpoint-related genes, and achieve a synergistic antitumor effect with immune checkpoint inhibitors.

We also investigated the expression of m6A-related genes (RBM15, FTO, YTHDF1, METTL3, YTHDC1, and YTHDF2) that significantly differed between the high-risk and low-risk groups. Some mRNAs are regulated by m6A, which is connected to cellular differentiation and cancer progression [49]. Conclusive evidence demonstrated that immunity in dendritic cells is regulated by m6A methylation by the protein YTHDF1 [50]. Our findings affect the m6A-related gene YTHDF1 and dendritic cells in immunity, and METTL3 enhanced the response to anti-PD-1 treatment [51], which suggests that these lncRNAs enhance the effect of tumor immunotherapy by regulating m6A-related target genes.

Our study developed and validated a new risk model for pyroptosis-related lncRNAs to explore the relationship between pyroptosis and COAD. However, according to the time-dependent ROC curve, the risk score model performed similarly to the classical staging and TNM models. The reason may be that ROC only considers the specificity and sensitivity of the method, and they cannot be considered equivalent in routine clinical practice. More experiments will be designed to authenticate the 9 pyroptosis-related lncRNAs model and clarify the mechanism by which pyroptosis-related lncRNAs regulate the pathological process of COAD.

## 5. Conclusion

We identified and verified a robust 9 pyroptosis-associated lncRNA signature prognostic risk model as an independent prognostic factor for COAD patients. A potential relationship with the tumor immune microenvironment and pyroptosis-associated lncRNAs suggested that these genes may be therapeutic targets for COAD. New immunotherapeutic drugs are expected to be developed by exploring these genes that trigger pyroptosis and are involved in the progression and tumorigenesis of COAD. Therefore, we recommend this 9 pyroptosis-associated lncRNA signature as a molecular marker to assess COA patients' prognostic risk.

## Data Availability

All data sources are obtained from The Cancer Genome Atlas (TCGA) database (https://portal.gdc.cancer.gov/) and processed by R software (version 4.0.4.).

## Supplementary Materials

Supplementary 1 Supplementary Figure 1: the expression of 9 pyroptosis-related lncRNAs between the high-risk and low-risk groups in human COAD tissues by RT-qPCR (delta Ct). ^∗^*p* < 0.05, ^∗∗^*p* < 0.01, and ^∗∗^*p* < 0.001.

Supplementary 2 Supplementary Table 1: the primer sequences for pyroptosis-related lncRNAs.
