# Supplementary material for: Construction and Validation of Pyroptosis-Related lncRNA Prediction Model for Colon Adenocarcinoma and Immune Infiltration Analysis
Source: Dis Markers. 2022 Sep 17;2022:4492608. doi: 10.1155/2022/4492608 (PMC9509522; doi:10.1155/2022/4492608)
Supplement: Supplementary 1 — Supplementary Figure 1: the expression of 9 pyroptosis-related lncRNAs between the high-risk and low-risk groups in human COAD tissues by RT-qPCR (delta Ct). ∗p < 0.05, ∗∗p < 0.01, and ∗∗p < 0.001. [file 4492608.f1.docx]

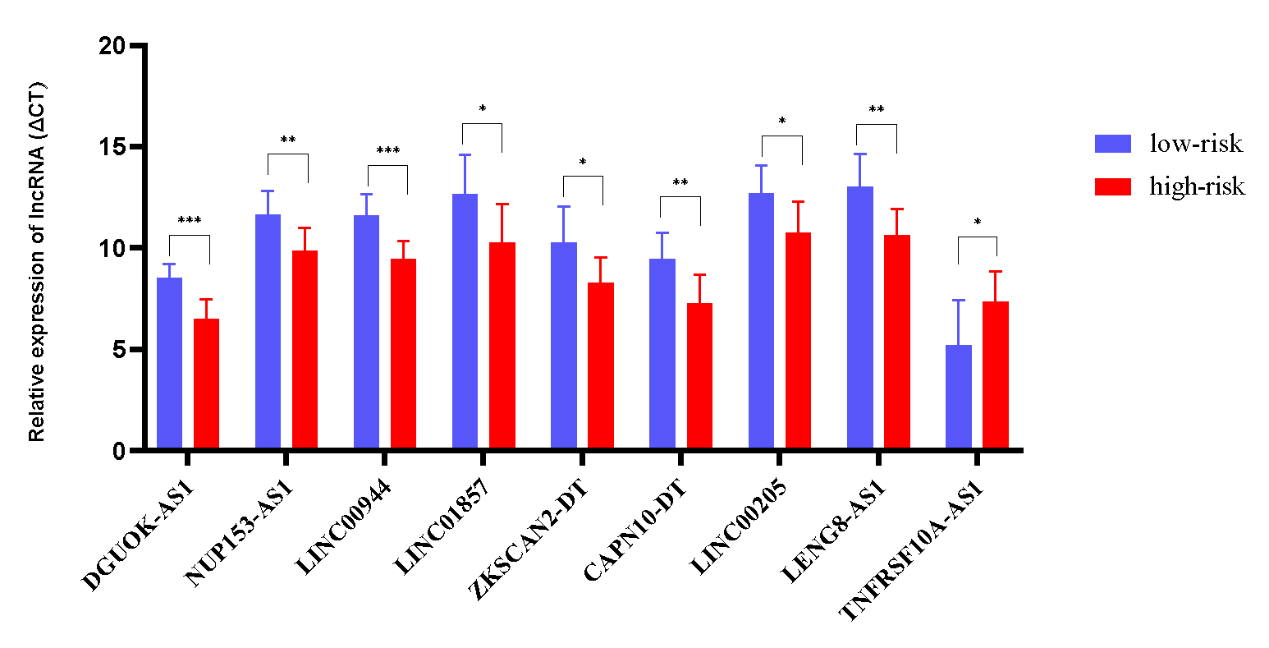


Supplementary figure 1. The expression of 9-pyroptosis-related lncRNAs between high-risk and low-risk group in human COAD tissues by RT-qPCR (delta Ct). *p < 0.05, **p < 0.01, **p < 0.001.
