# Supplementary material for: Construction and Validation of Pyroptosis-Related lncRNA Prediction Model for Colon Adenocarcinoma and Immune Infiltration Analysis
Source: Dis Markers. 2022 Sep 17;2022:4492608. doi: 10.1155/2022/4492608 (PMC9509522; doi:10.1155/2022/4492608)
Supplement: Supplementary 2 — Supplementary Table 1: the primer sequences for pyroptosis-related lncRNAs. [file 4492608.f2.docx]

## Supplementary Table 1: The Primer Sequences for Pyroptosis-related lncRNAs

| Primer_ID | Primer_seq | Primer_seq |
| --- | --- | --- |
| DGUOK-AS1 | 1Forward | GAAAGGGACAGGAAGCATAA |
|  | 1Reverse | AGAGTTCAGCAGCATCCAA |
| LINC00944 | 1Forward | ACGCACATCAGGAAGACAG |
|  | 1Reverse | TTGAGTTACAGGGACCGAAG |
|  | 2Forward | AGAAATGCTGGAAGAACGAG |
|  | 2Reverse | TGTTGATGGAGGACAGAGG |
| LINC01857 | 1Forward | TCTTCACTACCCTGTGGGA |
|  | 1Reverse | ACTGCTTGCCTTCTGTTCTT |
|  | 3Forward | GGACATAAGAACAGAAGGCAA |
|  | 3Reverse | GACTCCTACAGGGCCACTAA |
| LINC00205 | 1Forward | ACAAACTTCAGCGTCTCGTCT |
|  | 1Reverse | ACTCCCAACCTCCAGAACC |
|  | 2Forward | TCAGCATTCACTCCATCACA |
|  | 2Reverse | CCCGTATCTCACACAACCC |
| CAPN10-DT | 1Forward | TGTAGGTAGGAGGCAGGAAA |
|  | 1Reverse | GCAGATGGAGAGGATGAAGT |
|  | 2Forward | GGGATTGTAGGGAGATGAGAG |
|  | 2Reverse | GAGGCAGACAGGGTTGATT |
| LENG8-AS1 | 1Forward | CAGCACGGACTCTGATACAA |
|  | 1Reverse | TGACTGGCTTCTTCCTTCC |
|  | 3Forward | GACAGGATCATCAGGCAAA |
|  | 3Reverse | GGACTCTTGAACTTCCACCA |
| ZKSCAN2-DT | 2Forward | CTCTTGTCTGTCCGTTGTTG |
|  | 2Reverse | CTACCCTCCCACTTCTCCA |
|  | 3Forward | TGAAGACCTGGGATTGATAGA |
|  | 3Reverse | AAGAGCAGCCACCATAAGAC |
| NUP153-AS1 | 1Forward | AGAATGTCGTCACTCATCGG |
|  | 1Reverse | AGCCCTTCCTCTTTGCCTA |
|  | 2Forward | AACCTCTTCCCATTCTCCTTC |
|  | 2Reverse | CTGTCTGCTTACTTCCTGCCT |
| TNFRSF10A-AS1 | Forward | TAGGATGAGAGCTGCCCACT |
|  | Reverse | GGCCGTCCAGTAAGCTAAGG |

All the validation reports of these primers can be found in http://www.igenebio.com/.
